# You Aren't Always What You Eat

**DOI:** 10.1371/journal.pbio.1001000

**Published:** 2010-11-16

**Authors:** Robin Meadows

**Affiliations:** Freelance Science Writer, Fairfield, California, United States of America

**Figure:**
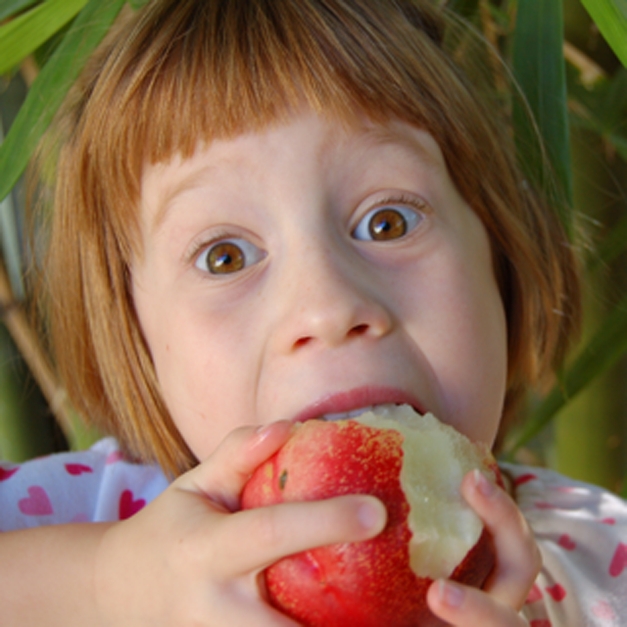
Nature or nutrition: what determines the menagerie of microbes that live in the guts of humans and their closest ape relatives?

The maxim “you are what you eat” goes only so far. The bacteria inhabiting our guts, which outnumber our own cells by perhaps 10 to 1, are commonly thought to reflect our diets. But other factors from geography to host physiology can also affect gut microbes, and sorting out their provenance is critical because they can affect our health for good or ill, from enhancing immune function to increasing the risk of stomach cancer. New research in this issue of *PLoS Biology* by Howard Ochman and colleagues counters the prevailing view that diet shapes the makeup of gut microbes, revealing that the host animal is a stronger determinant of these bacterial worlds in our digestive tracts.

On the face of it, gut microbe composition could be determined by either the host or its environment. Mammals are born with sterile digestive tracts and usually get their first gut microbes from their mothers, but thereafter acquire new ones from their environment. Moreover, there is evidence on both sides of the host versus environment debate. The evolutionary relationships of some gut bacteria are known to match those of their hosts, notably *Helicobacter pylori*, which is found in about half of people and is linked to stomach ulcers and cancer. However, work on great apes had suggested that diet is the major determinant of gut microbe composition.

To sort out whether gut microbe composition depends primarily on the host or its environment, Ochman and colleagues compared fecal samples from five closely related hominid species: Eastern and Western lowland gorillas, bonobos, and three subspecies of chimpanzees from several African countries as well as people from Africa and North America. By using fecal samples from locations separated by such considerable distances, the researchers effectively knocked out any effects of geography and the local environmental on gut microbes.

The researchers constructed two phylogenetic trees from the fecal samples: one of the great ape hosts, which was based on mitochondrial DNA, and the other of their gut microbe communities. Gut microbe diversity and abundance were based on small subunit ribosomal RNA genes, which are unique to bacteria and other prokaryotes. In turn, the “gut microbe” tree was based on the relative abundance of the various microbe species in each fecal sample, much as physical measures such as femur lengths from, say, mouse to elephant are used to infer evolutionary relationships.

The resulting gut microbe tree mirrored that for hominids; the patterns of relationships among the gut microbe communities were identical to those among the five great ape species. This congruence neatly establishes hosts as the major force behind the makeup of gut microbe communities. Further, the concordance of the two phylogenetic trees suggests that the gut microbe communities were shaped by divergences in host physiology over the course of great ape evolution.

The disparity with previous findings is partly because this new work entailed surveying many more fecal bacteria sequences per host, with a median of 28,000 sequences. In contrast, in previous studies only 100 to 200 bacterial sequences were surveyed per host, which is insufficient for gauging the diversity and abundance of species in the complex microbe communities living in our digestive tracts.

This work brings us closer to understanding the acquisition and evolution of the gut flora that can affect our health for better or for worse. Even though new microbes are constantly arriving in digestive tracts, their makeup is largely determined by the distinctive physiologies of people and our closest relatives. Gut microbe communities are thus predictable rather than variable, as they would be if determined largely by the host's external environment. Perhaps this is not altogether surprising, given that we hosts essentially are the environment for the worlds of enteric microbes within us. In this case at least, what we are trumps what we eat.

**Ochman H, Worobey M, Kuo C-H, Ndjango J-BN, Peeters M, et al. (2010) Evolutionary Relationships of Wild Hominids Recapitulated by Gut Microbial Communities. doi:10.1371/journal.pbio.1000546**

